# Synonymous Mutations in the Core Gene Are Linked to Unusual Serological Profile in Hepatitis C Virus Infection

**DOI:** 10.1371/journal.pone.0015871

**Published:** 2011-01-06

**Authors:** Agata Budkowska, Athanassios Kakkanas, Eric Nerrienet, Olga Kalinina, Patrick Maillard, Srey Viseth Horm, Geena Dalagiorgou, Niki Vassilaki, Urania Georgopoulou, Michelle Martinot, Amadou Alpha Sall, Penelope Mavromara

**Affiliations:** 1 Institut Pasteur, Hépacivirus et Immunité Innée, CNRS URA 3015, Paris, France; 2 Molecular Virology Laboratory, Hellenic Pasteur Institute, Athens, Greece; 3 Virology Laboratory, Pasteur Institute of Cambodia, Phnom Penh, Cambodia; 4 Molecular Microbiology-Virology Laboratory, Pasteur Institute of St. Petersburg, St. Petersburg, Russia; 5 INSERM, U-773, Centre de Recherche Biomédicale Bichat Beaujon CRB3, Université Paris VII, Hôpital Beaujon, Clichy, France; 6 Arbovirus and Hemorrhagic Fever, Pasteur Institute of Dakar, Dakar, Senegal; University Heidelberg, Germany

## Abstract

The biological role of the protein encoded by the alternative open reading frame (core+1/ARF) of the Hepatitis C virus (HCV) genome remains elusive, as does the significance of the production of corresponding antibodies in HCV infection. We investigated the prevalence of anti-core and anti-core+1/ARFP antibodies in HCV-positive blood donors from Cambodia, using peptide and recombinant protein-based ELISAs. We detected unusual serological profiles in 3 out of 58 HCV positive plasma of genotype 1a. These patients were negative for anti-core antibodies by commercial and peptide-based assays using C-terminal fragments of core but reacted by Western Blot with full-length core protein. All three patients had high levels of anti-core+1/ARFP antibodies. Cloning of the cDNA that corresponds to the core-coding region from these sera resulted in the expression of both core and core+1/ARFP in mammalian cells. The core protein exhibited high amino-acid homology with a consensus HCV1a sequence. However, 10 identical synonymous mutations were found, and 7 were located in the aa(99–124) region of core. All mutations concerned the third base of a codon, and 5/10 represented a T>C mutation. Prediction analyses of the RNA secondary structure revealed conformational changes within the stem-loop region that contains the core+1/ARFP internal AUG initiator at position 85/87. Using the luciferase tagging approach, we showed that core+1/ARFP expression is more efficient from such a sequence than from the prototype HCV1a RNA. We provide additional evidence of the existence of core+1/ARFP *in vivo* and new data concerning expression of HCV core protein. We show that HCV patients who do not produce normal anti-core antibodies have unusually high levels of antit-core+1/ARFP and harbour several identical synonymous mutations in the core and core+1/ARFP coding region that result in major changes in predicted RNA structure. Such HCV variants may favour core+1/ARFP production during HCV infection.

## Introduction

Hepatitis C virus (HCV) infection is a major cause of chronic liver disease worldwide, which can evolve to hepatic steatosis, cirrhosis and hepatocellular carcinoma [1]. HCV is an enveloped virus of the *Flaviviridae* family with a single-stranded positive sense RNA genome of about 9.6 kb. The viral genome is composed of a 5′non-coding region (UTR), one ORF encoding the precursor protein of about 3000 amino-acids, and a 3′UTR. The polyprotein is cleaved by host and viral proteases into the core protein, envelope glycoproteins E1 and E2, P7, and several non-structural proteins that have various functions in RNA replication and virus morphogenesis [2].

Bioinformatic analyses of the HCV RNA fragment that encodes the virus nucleocapsid protein provided evidence that this sequence is highly conserved and has a particularly complex structure, suggesting that it has multiple functions. Indeed, as for other human viruses such as HIV or HBV, HCV has an alternative open reading frame (ARF) that overlaps with a second gene, in this case that encoding core [3]. Experimental data confirmed that the HCV ARF can be expressed in cell-free systems [4], [5] and in mammalian cells [6], [7], [8]. The biological properties of this protein (known as ARFP, F or core+1 protein, but that will be referred here to as core+1/ARFP), its role in the HCV life-cycle and pathogenesis of infection remain elusive. The core+1/ARFP has not yet been detected either in patient sera or in infected tissues. However, unusual translation events resulting in core+1/ARFP production most probably do take place in natural HCV infection, as core+1/ARFP-specific humoral and T-cell responses have been detected in HCV-infected individuals [3], [4], [9], [10], [11], [12], [13], [14], [15].

Core+1/ARFP production could be initiated by one of several molecular mechanisms in cultured cells: (i) a ribosomal frameshift promoted by a cluster of 10 adenines at codons 8–11 of HCV RNA, which could induce either +1 or –1 programmed frameshift (limited to the genotype 1 genome), [4], [5], [7], [16]; (ii) a double ribosomal frameshift, with the first event occurring at codon 42 (in phase +1) and the second event at codon 144 (in phase -1), which enables “reframing” of translation and production of the “doubly frameshifted core protein” for genotype 1b HCV [17]; (iii) “transcriptional slippage” within the region of the 10 consecutive adenines at codons 8–11 of HCV-1 strains [18]; (iv) an internal translation initiation mechanism at codons 85–87[8] or codon 26 [6]. Notably, ribosomal or transcriptional slippage occurs in genotype 1a HCV only when 10 consecutive adenines are present in the core region, in codons 8–11[19].

Core+1/ARFP expressed *in vitro* is rather unstable and is readily degraded by the proteasome complex [5], [7], [20], [21]. Nevertheless, a perinuclear distribution of core+1/ARFP could be observed in transfected cells [7], [20]. Core+1/ARFP does not appear to play a role in virus replication *vitro* [22]. In addition, point mutations within the JFH-1 core+1/ARFP ORF or chimeric H77/JFH-1 strains do not affect virus production [16], [23]. However, the basic nature of core+1/ARFP, and the fact that it localises to the cell cytoplasm, suggests the possibility that it interacts with the HCV RNA [24].

The HCV core protein influences lipoprotein metabolism, cell transformation, regulation of cellular genes, transcriptional mechanisms, apoptosis, and cellular immune responses [25], [26]. It is conceivable that core+1/ARFP could be responsible for some of these biological functions attributed up to now to core. Indeed, the induction of fibrogenic chemokines [27], suppression of cellular p21 [28], perturbation of the microtubule network [29], activation of the intracellular NF-kB pathway[30] and enhancement of c-myc activity [14] have all recently been attributed to core+1/ARFP. These findings suggest that core+1/ARFP could play a role in HCV pathogenesis.

In this study, high levels of anti-core+1/ARFP antibodies were found in three patients from Cambodia, who had no antibodies directed against the core protein detectable by routine and home-made assays. This unusual serological profile could be linked to several synonymous mutations in the core and core+1/ARFP coding region that result in major changes in predicted RNA structure, and that may favour core+1/ARFP production. Our study provides further evidence for the production of core+1/ARFP in natural HCV infection and new information concerning the expression of the core protein, which is thought to play a major role in HCV pathogenesis.

## Materials and Methods

### Plasma samples

A panel of 87 plasma samples were from volunteer blood donors residing in Cambodia. All samples were collected between November 2002 and June 2003 by the National Blood Transfusion Centers (NBTC) in Phnom Penh and Battambang, in the Kingdom of Cambodia. Blood donations were from voluntary donors that included patient family members (“replacement donors”), unpaid volunteer donors from NBTC (“spontaneous donors”), and samples collected through mobile blood campaigns (“external donors”). All plasma samples were screened by NBTC for various serological markers and selected samples were coded before reaching the Virology Unit of the Pasteur Institute of Cambodia, where they were processed.

The panel included 87 plasma samples: 58 HCV positive plasma were selected by the NBTC among other samples found positive by routine diagnostic assays and completed with 29 HCV negative plasma collected from the same region.

### Ethics Statement

All plasma samples used in this study were from volunteer blood donors. Scientific agreement has been signed between the Pasteur Institute of Cambodia and NBTS concerning the improvement of HCV diagnostics. All samples were coded before reaching the Virology Unit of the Pasteur Institute of Cambodia, and thus treated anonymously. The protocol for the study has been submitted to the National Ethics Committee for Health Research at Ministry of Health of the Kingdom of Cambodia in September 2002 by the Pasteur Institute of Cambodia. Authorisation was granted by the National Ethics Committee for Health Research and agreement delivered by the Ministry of Health of the Kingdom of Cambodia in October 2002 (Document signed under No 1296/02 OGH).

### Serological analyses

Of 87 above mentioned plasma samples, 58 were found to be positive for anti-HCV antibodies using 3 assays: 1) Serodia® HCV (Fujirebio, Japan), 2) Monolisa anti-HCV PLUS Assay Version 2 (BioRad, Marne la Coquette, France), 3) a third generation microparticle enzyme immunoassay (MEIA) (AxSYM HCV, version 3.0; Abbott, Wiesbaden, Germany). Anti-HCV antibodies were also measured using a Chiron RIBA HCV 3.0 Strip Immunoblot Assay (SIA) (Ortho Clinical Diagnostics Illkrich, France).

HCV RNA was determined by PCR in the 5′ non-coding region (UTR). After reverse transcription, nested PCR targeting the 5′UTR was performed as previously described [31], [32]. The virus load in selected sera was measured by the b-DNA assay (Versant HCV RNA 3.0, Simmens Health Care Diagnostics St. Denis, France). HCV core antigen was assessed by commercial ELISA assay (Ortho Diagnostics, Wako, Germany).

### HCV genotyping

HCV NS5B region sequences in RNA isolated from plasma were amplified by nested RT-PCR [33]. cDNAs were obtained by reverse transcription in a 20 µl reaction mixture containing: 300 ng of random primer (hexanucleotide Pd(N)6, Roche), 10 µl RNA, 0.4x RT buffer (Promega), 0.5 mM dNTP, 24 U RNAsin (Promega), 8 U AMV RT (Promega). The 5′-TAT GAY ACC MGV TGY TTT GAM TC-3′ and 5′-GCN GAR TAY CTV GTC ATR GCY TC-3′ primers were used to amplify a 388 bp NS5B PCR product. The second round PCR primers: 5′-CGC TGY TTT GAM TCV ACN GTC AC-3′ and 5′-CTV GTC ATR GCY TCY GTR AAV CTC-3′ were used to amplify a 371 bp NS5B nested-PCR product. The 50 µl reaction mixture for the first PCR step contained 5 µl cDNA, each primer at 0.4 µM, 1.5 mM MgCl2, 0.2 mM dNTP and 1.5 U Tag DNA polymerase, the whole in 1x Tag DNA polymerase buffer. Nested-PCR, primed with 2 µl of PCR product, was performed as for the first PCR run. The NS5B-derived sequences were analysed and compared to reference sequences with the Phylip sequence analysis package (version 3.6 alpha 3) using DNAdist with Kimura's correction, followed by the neighbour-joining method [33].

### Construction of plasmids

Cloning was performed according to standard protocols (Maniatis), and all plasmid constructions were confirmed by DNA sequencing. cDNA fragments between nucleotides 1-873 that contain the core/core+1 coding sequences from the HCV1a H strain or from the Cambodian clinical isolate number 34 (HCV1a p34) were amplified from pHPI-1397 [33], [34] and the TOPO-based plasmid MR0582-1, using 5′-CTAG*CTAGC* **ATG**AGCACAAATCCTAA-3′ (sense) and 5′-GC*TCTAGA* **TCA**GGCTGAAGCGGGC-3′ (antisense) primers (here, and in following primers, restriction sites are shown in italics, and initiator ATG and termination codons are in bold) and were blunt-end ligated into the *Hinc*II site of pUC19 to yield pHPI-8153 and pHPI-8156, respectively. To express the core-coding region in eukaryotic cells, the *Nhe*I-*Xba*I fragment from these plasmids was cloned into the pCI eukaryotic expression vector to yield pHPI-8161 (HCV1a H) and pHPI-8162 (HCV1a p34).

For the synthesis of recombinant genotype 1a core protein in *E.coli*, a cDNA fragment between nt 1 and 360 was amplified from pHPI-1319[34] using 5-GGAATTC*CAT* ***ATG***AGCACGAAT-3 (HCV1a sense) and 5′-CCC*AAGCTT*ACCCAAATTGCG-3′ (antisense) primers, and blunt-end ligated into the *Hinc*II site of pUC19 to yield pHPI-8128. Next, the *Nde*I-*Hind*III fragment from pHPI-8128 was cloned into the pET20b (+) expression vector, upstream of the hexahistidine tail, to generate plasmids pHPI-8130. To express the HCV1a core+1/ARFP coding region in *E.coli*, a cDNA fragment between nt 44 and 487 was amplified using 5′-CATGCCATGGC**ATG**GCCAACCGTCGCCCACA-3′ (sense) and 5′-CCCAAG CTTGGGTCCCGCCGTCTTCCAGAACCCGGA-3′ (antisense) primers, and the *Nco*I–*Hind*III digested PCR product was cloned into the pET20b(+) expression vector.

To construct the IRES-ARF-LUC expression cassette, a cDNA fragment from HCV nt 1 to 484 was amplified from pHPI-8162 (HCV1a p34) or pHPI-8161 (HCV1a H) using 5′-C*GGATCC* **ATG**AGCACGAATCCTAAACC-3′ (sense) and 5′-C*GGATCC*TCGCCGTCCTCCAGAACCCGGAC-3′ (antisense) primers, and cloned into the *Sma*I site of pUC19, to give pHPI-8217 and pHPI-8221. Next, the *Bam*HI-*Bam*HI fragment from these plasmids was cloned directly into pGEM-*luc* (Promega), in frame with the luciferase gene, to yield plasmids pHPI-8224 (HCV1a p34 ARF-LUC) or pHPI-8223 (HCV1a H strain ARF-LUC). Finally, the *Xho*I-*Xho*I fragments of these plasmids were replaced into pHPI-1808 (kindly provided by M. Kochlios; based on the pEGFP N3 expression vector, that contains the C-terminal ARFP sequence fused to GFP) to construct pHPI-8227 and pHPI-8235 that contain the IRES-ARF-LUC sequences inserted into eukaryotic expression vector. Plasmid pHPI-8234 was also constructed, which contains the *Xho*I fragment of HCV1a (H strain) in the reverse orientation.

### Amplification, cloning and sequencing of the HCV core gene

Total RNA extracted from the plasma of 8 patients infected with genotype 1a HCV (5 with normal anti-core responses and 3 who were negative for anti-core and highly positive for anti-core+1/ARFP*) w*as reverse transcribed. Then, the full-length core gene was amplified by PCR using 5′CTTGTGGTATGCCTGATA3′ and 5′ACGATGCTGGWGTTRGGGCA3′ primers, followed by nested PCR with 5′-GAGGTCTCGTAGACCGTGCA-3′ and 5′GGCAATCATTKGTRAGATGGTA3′ primers. The PCR products were cloned into a plasmid (Topo TA cloning, Invitrogen) and sequenced in triplicate. Nucleotide and protein sequences were aligned using the CLUSTAL W program. Predicted analyses of RNA secondary structures in the core and core+1/ARFP coding regions were performed using the mfold program (version 3.2). http://frontend.bioinfo.rpi.edu/applications/mfold/cgi-bin/rna-form1.cgi.

### Expression and purification of the HCV core and core+1/ARF Proteins

Expression and purification of the histidine-tagged HCV core+1/ARFP and core protein aa(1-120) was carried out as previously described [34], [35]. It is important to note that the recombinant core+1/ARFP antigen deliberately does not contain amino acids from the N-terminal part of the core protein. Furthermore, tagging the core+1/ARF at the C-terminal end appears to minimize the possibility that mosaic core/core+1/ARFP proteins are obtained due to the frameshifting events that are favoured in E.coli (N.Vassilaki and P.Mavromara unpublished data). The size and identity of the recombinant proteins were controlled by Western blot analysis using anti-core+1/ARFP and anti-core antibodies.

### Detection of anti-core and anti core+1/ARFP antibodies by ELISA

Antibodies to HCV core protein were detected using a synthetic core peptide aa(3–75) of genotype 1a and recombinant core protein aa(1–120). The assay conditions were set-up using a reference panel of human sera, which included 10 HCV-positive, 10 HBV-positive samples, and 10 plasma from healthy controls.

Antibodies directed against core+1/ARFP were detected using a recombinant 1a protein. Rabbit anti-ARFP antibodies [20] and 5 serum samples from HCV-infected patients that were positive for anti-core+1/ARFP and rabbit anti-core+1/ARFP antibody were used as positive controls. The specificity of the assay was confirmed using anti-core+1/ARFP positive samples kindly provided by J.M Pawlotsky.

The antigens were used at a concentration of 1–5 µg/ml. Plasma samples were tested diluted 1∶20 in 0.1% BSA in PBS/Tween. The cut-off value was determined as the mean of the absorbance values recorded for negative samples, and samples were considered positive if the ratio of the tested/mean negative values was >2.1. A panel of HCV-negative sera from France and Cambodia was used to provide negative controls.

The presence of anti-core+1/ARFP antibodies in plasma samples negative for anti-core antibodies was confirmed using ELISA test with synthetic core+1/ARFP peptide encompassing aa sequence 91–106 of the protein, as previously described [4].

### Cell transfection and luciferase assay

The Huh-7 cell line was kindly provided by R. Bartenschlager and BHK-21 and HeLa cell lines by B. Roizman. All cell types were initially purchased in ATCC Cell Biology Collections. Cells were cultured in standard conditions, and transfected using JetPEI transfection reagent (Polyplus). Luciferase activity was measured with a Glomax 20/20 luminometer [16].

### Western blot

Cells were solubilized using the Triple Detergent Reagent supplemented with a protease inhibitor cocktail (Roche) and 0.07 mM PMSF and separated by 15% SDS-PAGE, transferred onto nitrocellulose membranes (Protran). For western blotting plasmas were used at a 1∶200 dilution, followed by HRPO-labelled anti-human IgG (Dako). Alternatively, rabbit antibodies to HCV core [34] and core+1/ARFP, and HRPO-labelled anti-rabbit IgG (Chemicon) were used.

## Results

### Patients with no detectable anti-core antibodies have a strong anti-core+1/ARFP response

**Figure 1:**
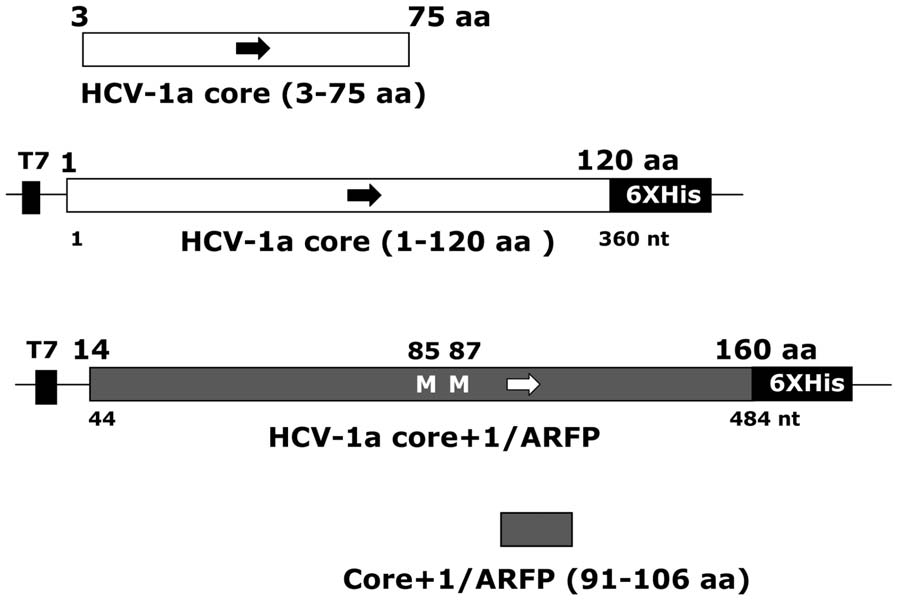
Schematic representation of the fragments of HCV core and core+1/ARF proteins used in ELISA for detection of anti-core and anti-core+1/ARFP antibodies. Core aa(1–120) and core+1/ARF aa(14–160) recombinant proteins were expressed in *E.coli* from the pET20T vector. Synthetic core aa(3–75) and core+1/ARF aa(91–106) peptides are shown. Details concerning the synthesis and purification of antigens are described in the Material and Methods section.

We analysed the prevalence of antibodies directed against HCV core and core+1/ARF proteins in a group of patients from Cambodia. The panel included 58 HCV-positive plasma samples (28 positive for genotype 6 HCV, 21 for genotype 1b, 8 for genotype 1a and 1 for genotype 2) and 29 plasma control samples, also from Cambodia, negative for HCV antibodies when assessed by routine ELISA and for HCV RNA by PCR. ELISA assays were developed, using recombinant proteins and synthetic peptides issued from HCV core and core+1/ARF proteins (Figure 1).

**Figure 2:**
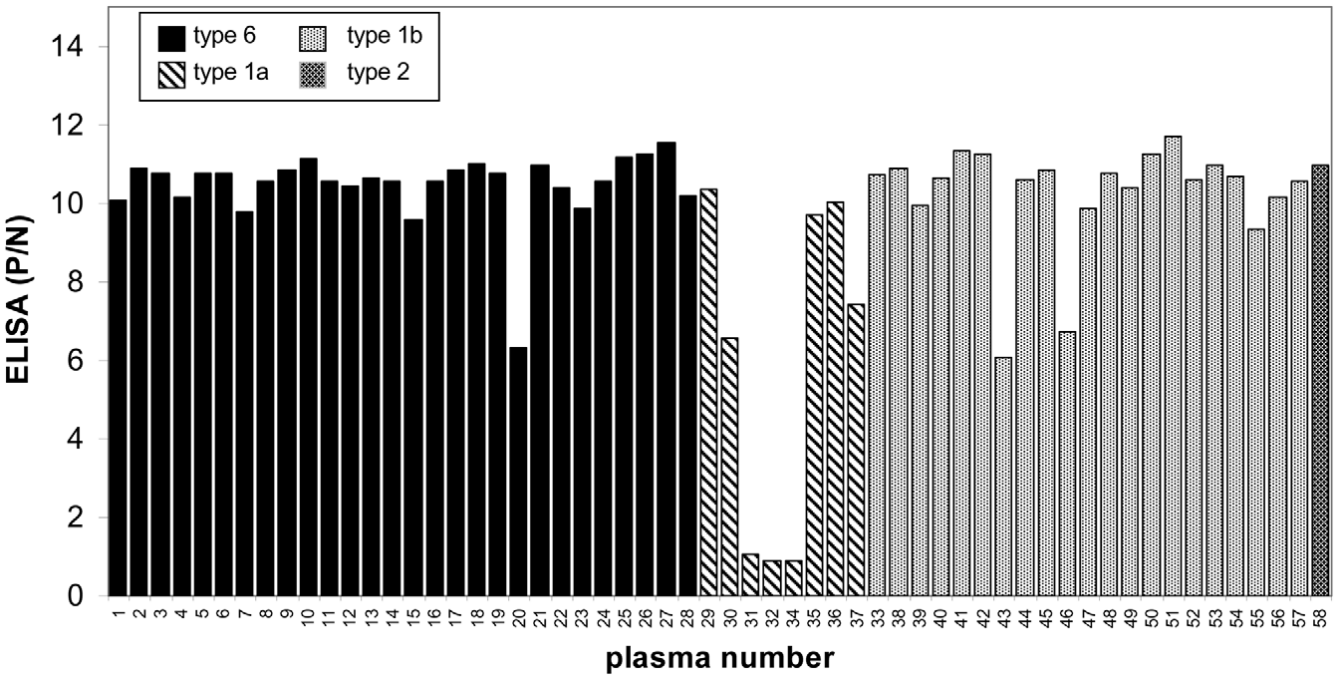
The prevalence of anti-core antibodies in a panel of 58 HCV-positive plasma samples from Cambodia, detected by ELISA based on core synthetic peptide aa(3–75). The panel included 28 plasma samples from individuals infected with HCV-6; 21 HCV-1b; 8 HCV-1a; and 1 HCV-2a samples. Twenty-nine HCV-negative samples from individuals living in Cambodia were included in the panel as negative controls. Three of 58 plasma samples (all from HCV-1a infected individuals, designated as p31, p32 and p34) were found negative for anti-core antibodies.

**Figure 3:**
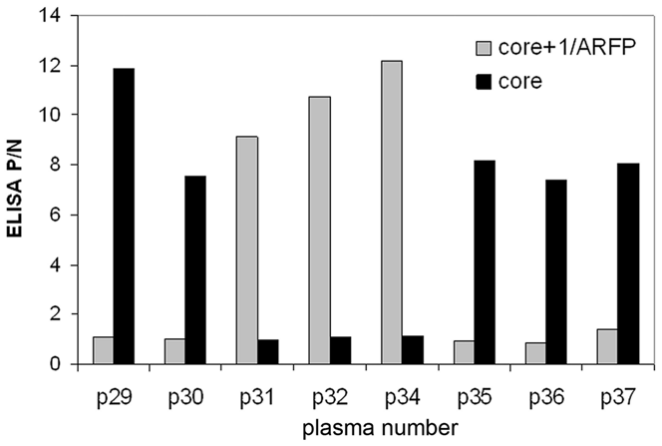
Detection of anti-core and anti-core+1/ARFP antibodies in plasma of HCV-1a genotype. Lack of anti-core antibodies in p31, p32 and p34 plasma samples by ELISA based on core aa(1–120) antigen correlated with strong anti-core+1/ARFP responses detected using ELISA based on core+1/ARF aa(14–160) protein.

Most plasma samples from HCV-infected patients (55/58) were positive for anti-core antibodies when tested using a synthetic core peptide aa(3–75) (Figure 2). However, 3 patients (designated p31, p32 and p34) infected with genotype 1a HCV showed negative for anti-core antibodies. This observation was confirmed using HCV core protein aa(1–120) (Figure 3).

The plasma samples from the panel were tested in parallel for their reactivity with recombinant core+1/ARFP. Anti-core+1/ARFP antibodies were detected in 8 serum samples; 5 of them were from patients infected with genotype 6 HCV and 3 from patients infected with genotype 1a HCV. Significantly, the highest levels of anti-core+1/ARFP antibodies were detected in the 3 plasma samples from patients infected with HCV 1a and lacking anti-core antibodies (Figure 3). The presence of anti-core+1/ARFP antibodies in these plasma samples was confirmed using ELISA test with synthetic core+1/ARFP peptide encompassing aa sequence 91–106 of the protein (data not shown).

Further analyses carried out using the Chiron RIBA HCV 3.0 Strip Immunoblot Assay (SIA) confirmed the absence of antibodies directed against the core protein (Table 1). This third generation semi-quantitative RIBA assay measures antibodies directed to core antigen c22p, synthetic peptide aa(10–53) and non structural proteins: NS3 c33c recombinant protein aa(1192–1457), NS4 peptides c5-1-1 aa(1694–1735) and c100p aa(1920–1935) and NS5 recombinant protein (NS5r) aa(2054–2995). Proteins c33 and NS5r are produced as fusion proteins with human superoxide dismutase. The intensity of the coloured bands is proportional to the amount of bound antibody and graded from 0 to 4+ [36], [37].

**Table 1:** Serological profiles in Cambodian patients infected with genotype 1a HCV.

SampleNumber	Viral load	c-100(NS4)	c-33(NS3)	C22(core)	NS5	Anti-coreaa(1-120)	Anti-core+1/ARFP	Core Ag(fmol/L)
p29	8 065 471	0	4+	4+	4+	+	-	6300
p30	342 966	4+	4+	3+	0	+	-	5200
p31	177 463	4+	4+	0	0	-	+++	-
p32	384 746	4+	4+	0	0	-	+++	690
p34	4 895 775	4+	4+	0	0	-	+++	5800
p35	12 091	0	3+	2+	0	+	-	-
p36	85 589	0	4+	4+	0	+	-	-
p37	15 069 598	0	0+	4+	0	+	-	7200

The absence of antibodies directed to epitopes localized in the immunodominant region of core aa(1–120) in plasma samples designated as p31, p32, p34 was confirmed using Chiron RIBA HCV 3.0 Strip Immunoblot Assay (SIA) (Novartis Vaccines and Diagnostics, Inc., Emeryville, CA). Virus load in all these plasma samples of 1a genotype was determined by b-DNA assay (Versant HCV RNA 3.0, Siemmens Health Care Diagnostics St. Denis, France) and the presence of HCV core protein was determined by a commercial ELISA assay Ortho Diagnostics (Wako, Germany).

Although no anti-NS5 antibodies could be detected for patients p31, p32 and p34, all tested positive for NS4 and NS3 antibodies, in addition to the strong anti-core+1/ARFP responses. Thus, the lack of anti-core antibodies was not due to a general defect of the humoral immune response in these patients.

Different levels of HCV core antigen were detected in these plasma samples. Patient p34 was strongly positive, p32 weakly positive for HCV core antigen, whereas p31 was found negative. These results correlated with the viral load, since p31 contained the lowest levels of the virus, and core antigen assay is considered less sensitive that detection of virus RNA by the b-DNA assay.

### Analysis of the core region of HCV isolates derived from the anti-core antibody negative patients

**Figure 4:**
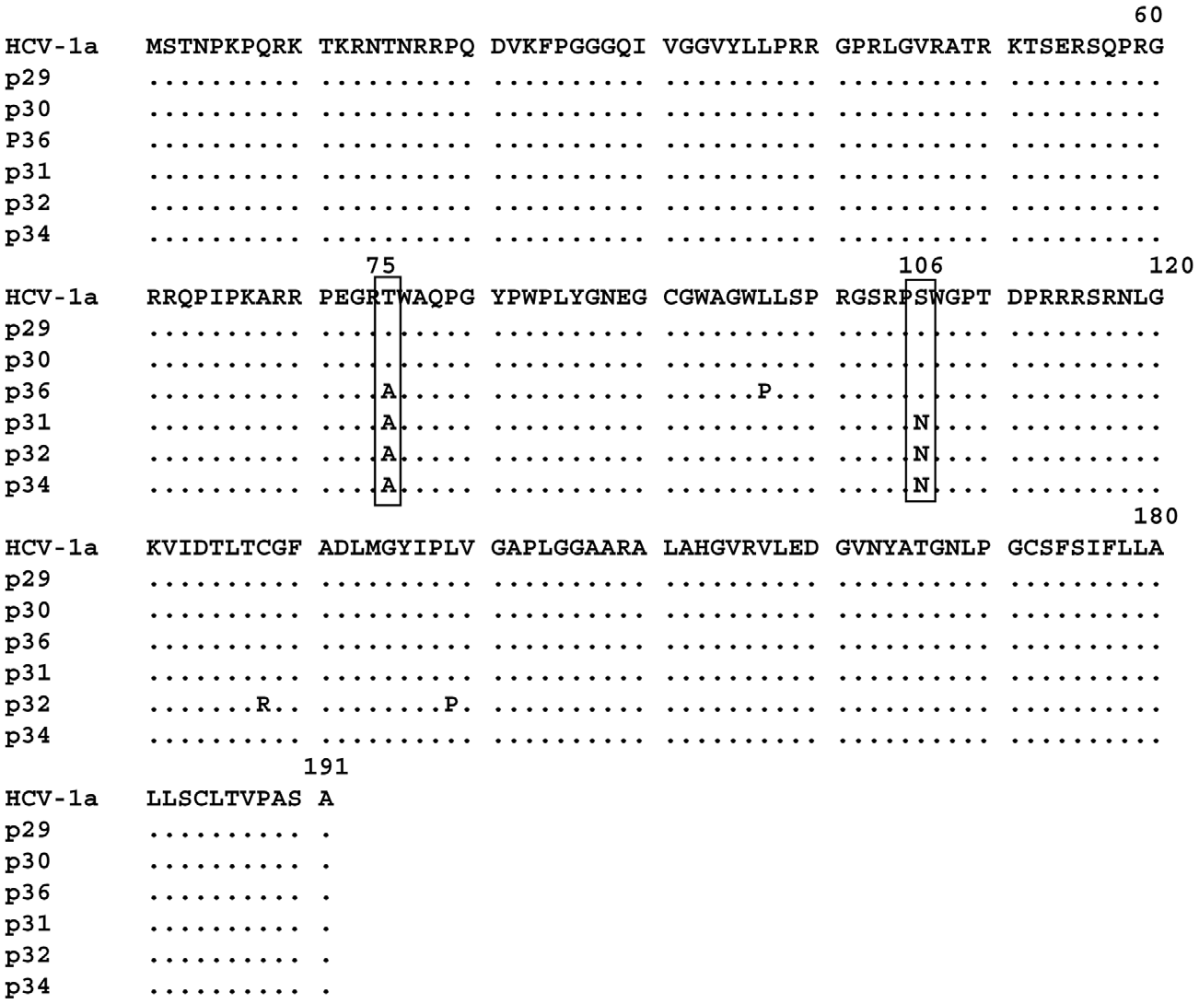
Amino-acid sequence alignment of the core region derived from three HCV-1a isolates positive for anti-core antibodies (p29, p30, p36) and those that were negative for anti-core antibodies (p31, p32, p34). cDNA fragments from the HCV core region were obtained and cloned into the TOPO TA vector as described in Material and Methods. Three clones were sequenced for each patient and all three were identical for each patient. Protein sequences were analyzed using the CLUSTAL W program. The amino-acid sequence of the HCV-1a H reference strain is shown.

We investigated further whether the absence of anti-core antibodies in the three patients described above was associated with particular mutations in the core gene. The nucleotide and amino-acid sequences of the core region derived from the three anti-core negative patients (p31, p32, p34) as well as three anti-core positive patients of genotype 1a (p29, p30, p36) were analysed. HCV RNA was extracted from plasma, amplified, and cloned into an expression vector. Three clones per sample were sequenced. Amino-acid and nucleotide sequences were aligned using the CLUSTAL W program. As shown in Figure 4 the core ORF was intact and a high amino-acid sequence homology was observed in all plasma samples analysed as compared with a consensus genotype 1a HCV core sequence. No substitutions in the aa (9–11) region were detected that could explain a more efficient initiation of synthesis of core+1/ARFP from this site [19]. Two amino acid changes T>A at aa75 and S>N at aa106 were found. However, similar substitutions have already been reported that were not associated with the absence of anti-core antibodies [38].

**Figure 5:**
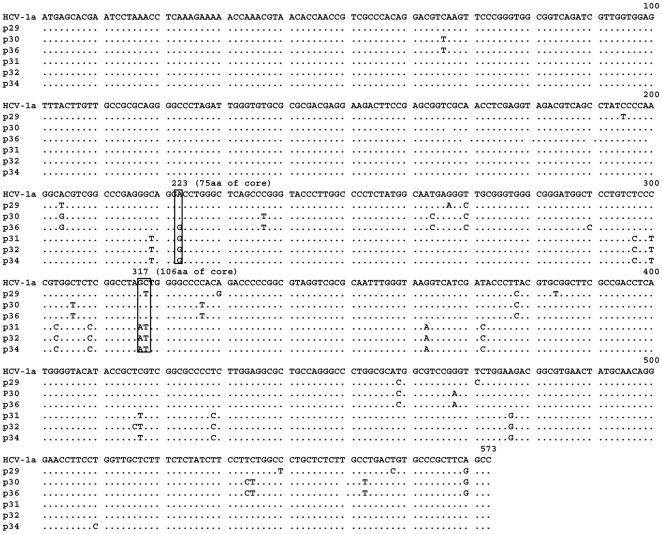
Nucleotide sequence alignment of the cDNA corresponding to the HCV core protein region shown in **Figure 4**. Non-synonymous mutations at nucleotides 223 and 317 are boxed; 10 synonymous mutations specific for the three core-antibody negative patients (p31, p32, p34) were observed. Nucleotide sequences were analyzed using the CLUSTAL W program.

Nevertheless, several nucleotide mutations were found in the core region of these patients as compared to a consensus sequence issued from genotype 1a HCV or the Cambodian HCV1a patients with normal core antibody levels (Figure 5). Two C>T and A>G transition mutations, situated at nucleotides 223 and 317 give rise to the amino acid changes cited directly above. Other mutations in the core region are synonymous and most of them (7/10) are positioned in the region encompassing aa(99–124). All synonymous mutations concern the third base, 5 represent a T>C mutation, 3 a C>T, 1 G>A, and 1 A>G. Interestingly, all three core antibody negative patients harboured identical mutations.

**Figure 6:**
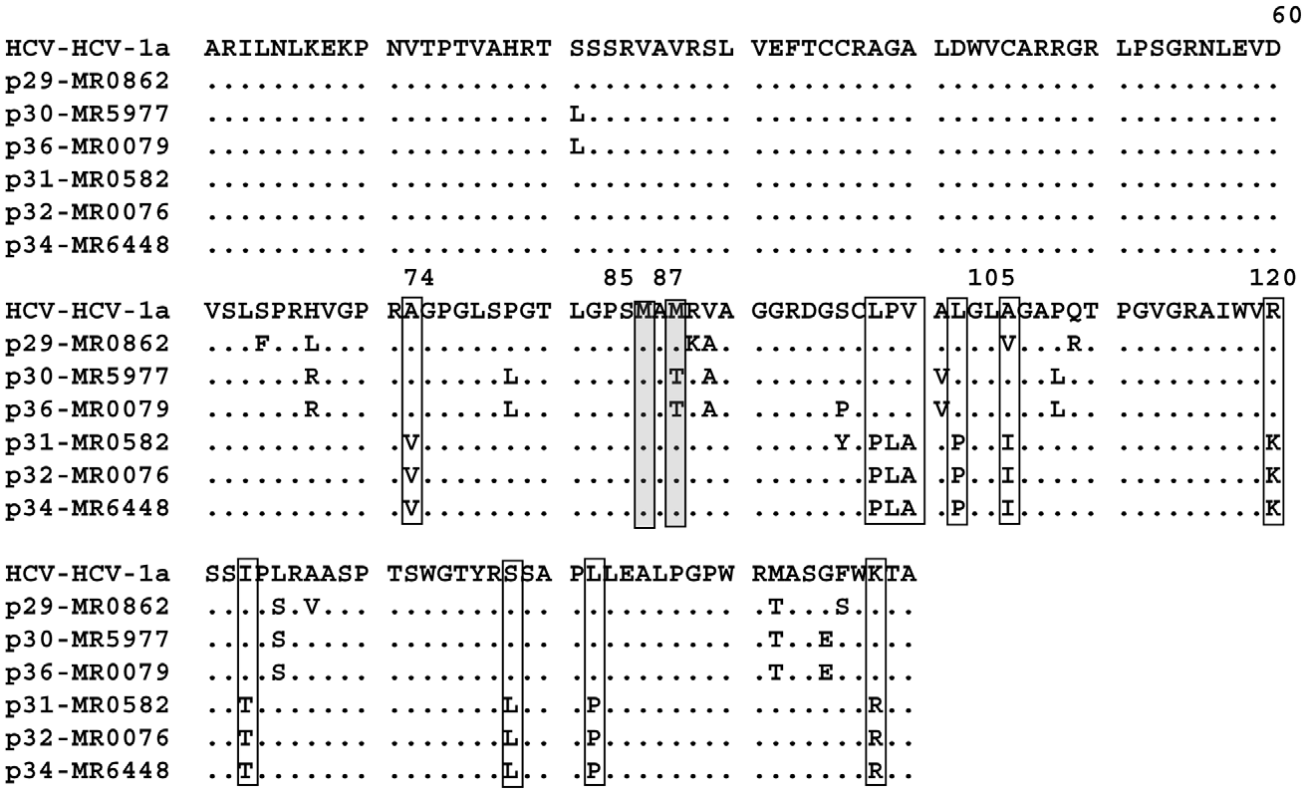
Deduced core+1/ARFP amino-acid sequences from cDNA shown in **Figure 5**. The sequences relate to isolates positive for anti-core antibodies (p29, p30, p36) and the three HCV-1a isolates negative for anti-core antibodies (p31, p32, p34). The corresponding amino-acid sequence of the consensus genotype 1a HCV is also shown.

In the deduced core+1/ARFP amino-acid sequence (Figure 6), several changes were found in p31, p32 and p34 samples and all of these were situated in the region corresponding to aa(73–158).

### The core region of the HCV isolates derived from the anti-core antibody negative patients is functional *in vitro*

**Figure 7:**
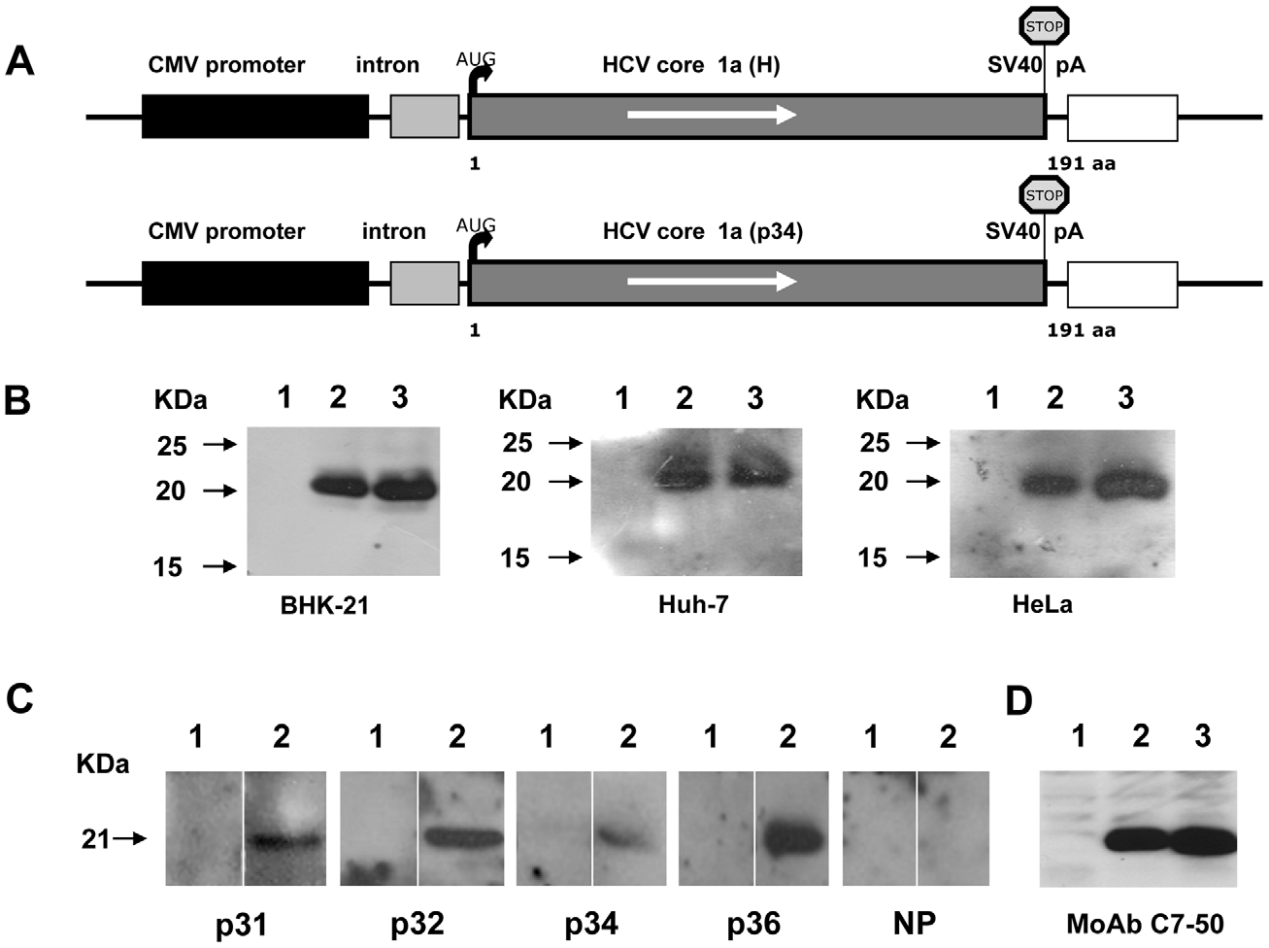
Expression of the HCV core protein from an anti-core negative patient (p34) in mammalian cells. (A) Schematic representation of constructs used for the eukaryotic expression of core from the p34 HCV-1a isolate using plasmid pHPI8162 and HCV-1a H strain using plasmid pHPI 8161 (described in Material and Methods). An artificial stop codon was inserted at the 3′ end of the core gene (STOP). (B) Transient expression of the cloned HCV core gene in cultured cells. Cells were transfected with pHPI 8161 or pHPI 8162 plasmids, or the pCI empty vector. After 48 hours, cell lysates were analyzed by Western blotting using rabbit anti-core antibodies. Lane 1: cells transfected with pCI empty vector; lane 2: with a plasmid encoding HCV core (H strain); lane 3: with a plasmid encoding HCV core p34. The 21 kDA HCV core protein was detected in of all cell types. (C) The expressed core protein (M.wt. 21 KDa) is reactive with all three unusual plasma samples, p31 p32 and p34 and with a control plasma p36 positive for anti-core antibodies in all assays. NP –HCV negative plasma; extract from cells transfected with pCI control plasmid (Lane 1) and from cells transfected with a plasmid encoding HCV core p34 (Lane 2). (D) The expressed core protein is reactive with anti-core MAbC7-50 that recognizes an epitope localized in the aa(21–57) sequence. Extract from cells transfected with the pCI control plasmid (Lane 1), with a plasmid encoding HCV core from H strain (Lane 2) and with a plasmid encoding HCV core from p34 (Lane 3).

It was thus interesting to see whether in spite of numerous mutations, the core region of HCV RNA could be expressed in mammalian cells. The core-coding region from one of the anti-core negative patients was cloned into a eukaryotic expression vector (Figure 7A) and transiently expressed in mammalian cells: BHK-21, Huh7 and HeLa. This resulted in the production of the 21kDa core protein that was recognised by anti-core MAbs specific for the N-terminal part of core (Figure 7D). This protein was also reactive with the three plasma found negative for anti-core antibodies by ELISA and routine assays (p31, p34, p32) and with plasma positive for anti-core antibodies (p36). These results demonstrated that unusual anti-core antibodies might be present in these three plasma samples, as they failed to recognise core antigens derived from the immunodominant N-terminal part of the protein. These antibodies were weak and detectable only by Western Blot with full length core protein. These data suggest that anti-core antibodies in these plasma samples were probably directed against the C-terminal part of the core protein, downstream from aa120.

**Figure 8:**
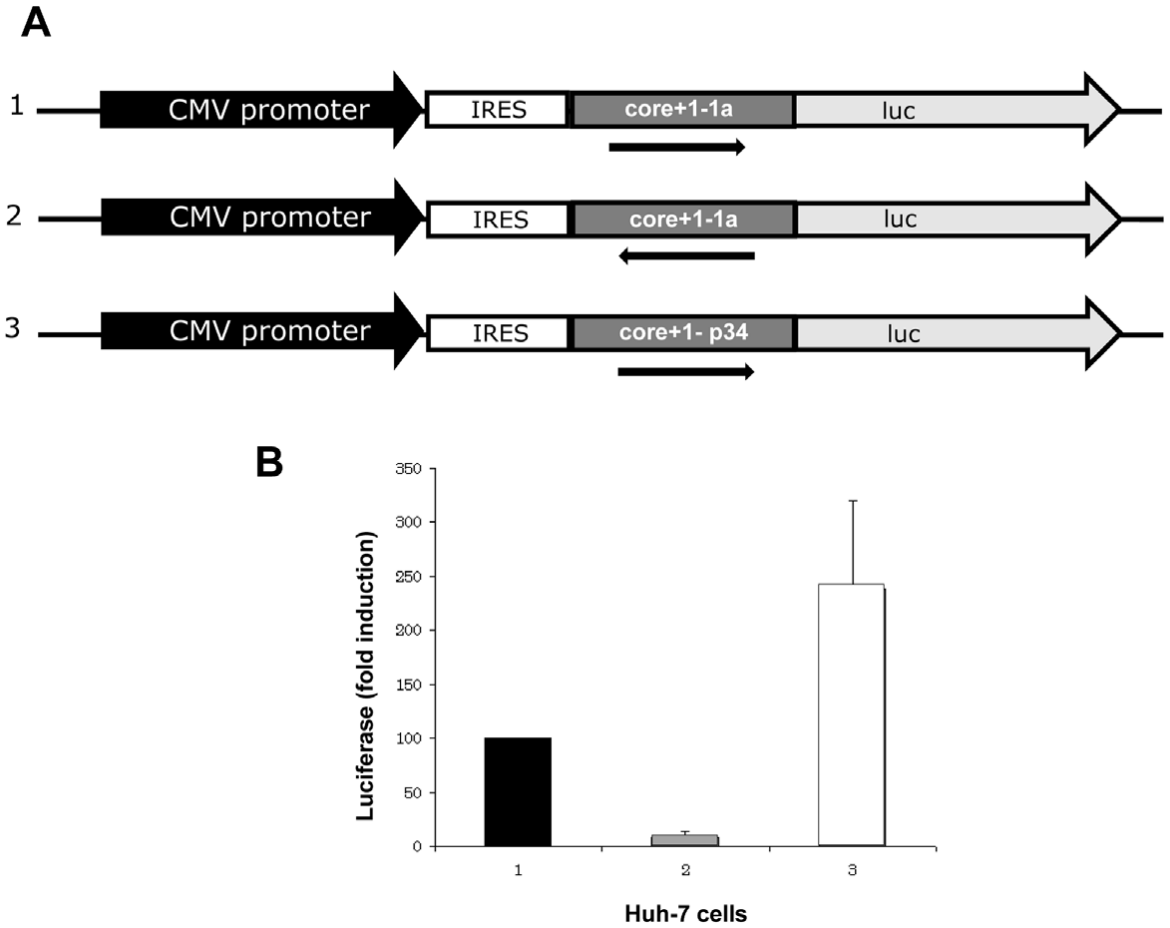
Expression of LUC-tagged core+1/ARFP construct from plasma p34 in Huh-7 cells. (A) Schematic representation of the Luc-tagged constructs. Core+1/ARFP coding sequences were fused in frame with the firefly Luciferase gene, under the transcriptional control of the CMV promoter and the translational control of the HCV-1a IRES. 1: core+1/ARFP from HCV-1a H strain (pHPI8234), 2: anti-sense core+1/ARFP construct from HCV-1a H strain pHPI8235 (negative control, see arrow), 3: core+1/ARFP from patient p34 (pHPI8227).(B) Huh-7 cells were transfected with each plasmid and the relative Luciferase activity derived from each construct were determined. The LUC levels were arbitrarily set at 100% for pHPI 8234. Values plotted correspond to the mean obtained in three separate experiments carried out in duplicate. Error bars represent standard deviation. Similar results were obtained using HeLa and BHK-21 cells.

We also examined the expression of core+1/ARFP *in vitro* from RNA of one of the three particular patients, using the luciferase tagging approach [8], since untagged core+1/ARFP is very unstable in transfected cells [5], [7], [20], [21]. Transient expression of core+1/ARFP-LUC was observed and the levels of transfection in Huh7 cells (Figure 8B), but also in BHK-21 and HeLA cell lines (not shown), was consistently (at least 2.5 fold) higher than that from a prototype 1a HCV RNA.

These results provided evidence that the core gene of the anti-core antibody negative patients is fully functional, as it efficiently supports the synthesis of both HCV core and core+1/ARF proteins in transfected cells. Furthermore, these observations demonstrated an advantage when core+1/ARFP is expressed from this sequence compared to that of the prototype HCV-1a. These observations suggest that the expression of core+1/ARFP may also be favoured from this mutated RNA during natural HCV infection.

### Analyses of HCV RNA secondary structure

Synonymous mutations could affect the stability and secondary structure of RNA. Especially an enrichment in cytosine bases at synonymous sites might have an effect on RNA stability [39]. Therefore, differences in the structure of HCV RNA in the core-coding region could play a role in the initiation of translation from internal codons or in frame-shift events. Thus, prediction analyses of the HCV RNA secondary structure were carried out using the m-fold computational program. The entire core region was used to predict the effect of mutations on the HCV RNA secondary structure.

**Figure 9:**
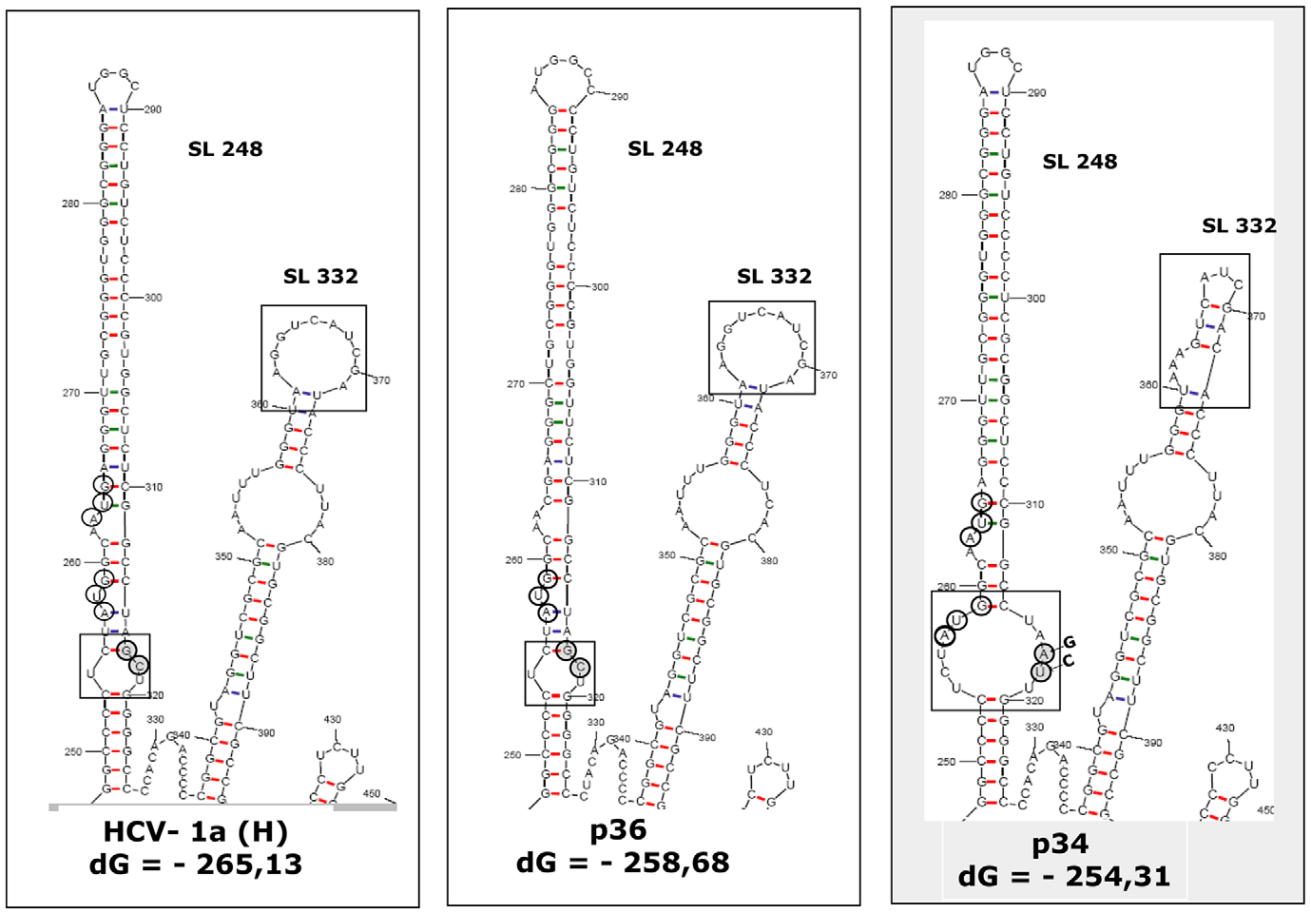
Prediction of stem-loop 248 and stem-loop 332 RNA folds, for HCV-1a H strain, the p36 isolate (positive for anti-core and negative for anti-core+1/ARFP antibodies) and p34 (negative for anti-core and positive for anti-core+1/ARFP). RNA secondary structure was predicted using the m fold program. Open boxes indicate structure alterations in the internal loop of stem-loop 248 and in the apical loop of stem-loop 332. Open circles represent the locations of internal translation initiation codons (AUG 85 and 87) that serve for core+1/ARFP synthesis. Gray circles represent the nucleotides that are altered in isolates from anti-core+1/ARFP antibody positive patients.

Strikingly, major differences were seen in SL248 when serum samples with the unusual serological profile were considered, compared with the prototype H strain of genotype 1a (Figure 9). In particular, GC>AT changes at nucleotides 317 and 318 alter the RNA structure by enlarging a bulge that brings AUG-85 from its original position at a stem into the loop structure. Such changes were not found in three RNA samples from the panel of HCV-1a genotypes with normal serological profiles. Additional mutations in SL337 might involve a G>A change at nucleotide 363, and T>C at 372, reducing the size of the upper terminal loop.

Altogether, the prediction analysis of HCV RNA revealed important conformational changes for patients with high anti-core+1/ARFP antibody responses within the core-coding region stem-loop structure that contains the core+1/ARFP initiator AUG-85 codon. It is intriguing to speculate that these changes could favour the initiation of core+1/ARFP synthesis in these patients.

## Discussion

The HCV core gene is more conserved than is necessary to conserve the encoded protein sequence [3], suggesting other functions for the virus RNA. Indeed, a second protein encoded by the core region of the genome has been discovered, designated here as core+1/ARFP (for review, see [24], [40]). The role of core+1/ARFP in the virus cycle and in the pathogenesis of HCV infection remains obscure, and this protein is not required either for virus replication or for production of infectious HCVcc (JFH1) in cell culture systems [16], [23]. Nevertheless, several studies have provided indirect evidence that core+1/ARFP is effectively produced during the natural HCV infection [9], [27].

In this study we have investigated the presence of anti-core and anti-core+1/ARFP antibodies in plasma samples from HCV-infected individuals living in Cambodia. Anti-core+1/ARFP antibodies were detected in 8/58 serum samples; 5 individuals were infected with genotype 6 HCV and 3 with genotype 1a. Generally, the levels of anti-core+1/ARFP antibodies (P/N values) were relatively low, and in six such cases anti-core antibody responses were normal. However, 3 patients infected with genotype 1a HCV developed exceptionally strong anti-core+1/ARFP responses and had no detectable antibodies directed towards the N-terminus of core.

**Figure 10:**
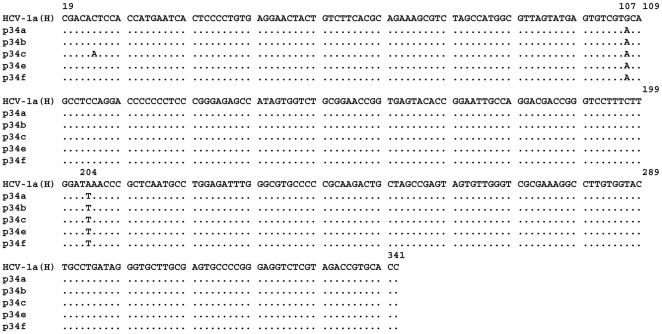
Nucleotide sequence alignment of the cDNA sequence that corresponds to the 5′UTR region from patient p34, negative for anti-core and positive for anti-core+1/ARFP antibodies. The c-DNA fragments were obtained by PCR and cloned into the HincII site of pUC19 vector. Five clones were isolated and sequenced. The nucleotide sequence of HCV1a H strain is shown.

Analyses of the HCV RNA from these patients showed that the entire core region was present and was functional, since the cloning and expression of this RNA resulted in the production of full-length core protein in mammalian cells. Also, the 5′UTR of these isolates was intact, excluding the possibility of abnormal expression of the HCV polyprotein (Figure 10). Furthermore, analyses of the amino-acid sequence of core protein predicted from its gene sequence did not show any specific changes that could explain the unusual serological profile. The deduced core+1/ARFP amino-acid sequence in the +1 frame was extremely conserved between the three patients, but was considerably different from that of the prototype 1a HCV or from the sequence seen in other Cambodian patients infected with genotype 1a HCV.

Interestingly, the absence of “normal” anti-core antibodies in 3 patients correlated with several identical synonymous mutations in the core gene. Most of these (7/10) were positioned in the region aa (99–124), encoding the so called “short form” of core+1/ARFP [20]. There is increasing evidence that “silent” mutations that do not alter the amino-acid composition of a gene product can affect transcription, splicing, or mRNA transport. The synonymous mutations do not alter mRNA levels, but they could influence RNA stability and change the rate of protein synthesis and thus cause a protein to fold into a alternative structure [41], [42]. Thus, they would have phenotypic effects with clinically important consequences [42].

Prediction analyses showed that the mutations in the core gene induce conformational changes in the internal loop of SL248 and in the apical loop of SL332 of HCV RNA, compared to HCV-1a RNA from patients with a normal anti-core response. Since SL248 contains codons 85–87 implicated in internal initiation of translation and production of the core+1/ARFP [8], such conformational changes could affect translation initiation within the core-coding region and favour the synthesis of the core+1/ARFP. The analyses of cloned HCV RNA also showed that although the nucleotide sequence spanning codons 85–87 was not altered, nucleotide changes at position 317 (CC to AT) resulted in a conformational change of the RNA structure that shifts AUG-85 from a stem into an internal loop. Such a change may favour the initiation of translation at position 85. Indeed, we detected an increased level of core+1/ARFP-LUC expression in transiently transfected mammalian cells when the sequence contained these mutations (Figure 8).

Our findings suggest that the absence of detectable anti-core antibodies, and the production of a strong anti-core+1/ARFP response, can be related to specific nucleotide motifs or mutations in the core and ARFP/core+1-coding region. Thus, in patients harbouring RNA with such motifs/mutations, efficient internal translation initiation at AUG-85 probably leads to the production of higher levels of core+1/ARFP. Core+1/ARFP is considered to be a short–lived and very labile protein [5], thus it can also be hypothesized that core+1/ARFP bearing these alterations would be of increased stability in the core-antibody negative patients.

It is noteworthy that, although core+1/ARFP is conserved amongst different HCV genotypes, the length of core+1/ARFP appears to be genotype-specific: by database analyses the longest form of core+1/ARFP has been deduced to be that of the 1a genotype [5]. In agreement with this, the three Cambodian patients in question harboured genotype 1a HCV.

In our study, cDNA from these patients induced the synthesis of the full-length 21 kDA core protein upon cloning and expression in mammalian cells (Figure 4). In addition, different concentrations of HCV core antigen could be detected in plasma samples from each of the three patients. Thus, the HCV variants (p31, p32, p34) identified in this study are not deficient in core protein production, but the patients rather induce the unusual pattern of the immune response. Indeed, the patients with distinctive (and identical) mutations in the core gene sequence seem to produce anti-core antibodies directed to the less immunogenic, C-terminal part of the protein. The question remains as to the reason of production of such unusual anti-core antibodies in the three Cambodian patients. One possibility is that these patients could produce increased levels of so-called “mini-cores”, as shown in studies *in vitro* [43]. Indeed, the synthesis of short forms of core (8–14 kDa) that contain the C-terminal portion of the protein was demonstrated for several infectious HCV strains that replicate *in vitro* in hepatoma cell lines [43]. These “mini-cores” are synthesized by internal translation initiation from initiator codons in close proximity to AUG-85, the main initiation codon for core+1/ARFP. Although they lack the RNA-binding region and immunodominant core epitopes, the “mini-cores” seem to be important functional components of the HCV protein repertoire, as they might mediate some biological functions of core, such as binding to lipid droplets [43]. The viruses that produce mini-cores (genotypes 1 and 2) also express larger core proteins that could play a role in particle formation [43]. The core assay involves antibodies that recognise epitopes in both N-and C-terminal parts of core. Thus it can potentially detect both the full-length and mini-core proteins. It is tempting to speculate that « mini-core » and core+1/ARFP may share common regulatory elements for internal translation initiation and therefore there could be a link between “mini-core” and core+1/ARFP production *in vivo*.

In conclusion, we provide further evidence for the synthesis of core+1/ARFP protein during natural HCV infection. We also demonstrate that unusually high levels of anti-ARFP antibodies can be linked to a lack of normal anti-core antibody responses. The synthesis of core+1/ARFP in such patients might be favoured by synonymous mutations in the core coding sequence, situated close to AUG-85, which induce conformational changes in SL248 and SL337. Thus higher levels of core+1/ARFP production may be linked to the properties of the mutated HCV core. Since HCV core protein is thought to play a major role in HCV pathogenesis, it would be worthy to determine whether negative result in anti-core antibody assay (and/or increased levels of anti-core+1/ARFP) could identify patients harbouring mutated core sequences, and whether this unusual serological pattern is associated with a particular pathology during HCV infection.
