# Correction: Synonymous Mutations in the Core Gene Are Linked to Unusual Serological Profile in Hepatitis C Virus Infection

**DOI:** 10.1371/annotation/cfafce35-02ca-43df-9d06-3c22fc92d349

**Published:** 2011-01-13

**Authors:** Agata Budkowska, Athanassios Kakkanas, Eric Nerrienet, Olga Kalinina, Patrick Maillard, Srey Viseth Horm, Geena Dalagiorgou, Niki Vassilaki, Urania Georgopoulou, Michelle Martinot, Amadou Alpha Sall, Penelope Mavromara

There was an error in reference 18. The correct reference 18 is: Ratinier M, Boulant C, Combet C, Targett-Addams P, McLauchlan J and Lavergne JP. (2008) Transcriptional slippage prompts recoding in alternate reading frames in the hepatitis C virus (HCV) core sequence from strain HCV-1. J Gen Virol, 89: p. 1569-78.

